# Cerebral activation caused by dental sounds: a functional magnetic resonance imaging study

**DOI:** 10.1007/s10266-023-00898-7

**Published:** 2024-02-03

**Authors:** Hiroyuki Karibe, Michihiko Koeda, Yuichi Kato, Tomoko Hama, Satoshi Tanaka, Amane Tateno, Hidenori Suzuki, Yoshiro Okubo

**Affiliations:** 1 Department of Pediatric Dentistry, School of Life Dentistry at Tokyo, The Nippon Dental University, 1-9-20 Fujimi, Chiyoda-Ku, Tokyo, 102-8159 Japan; 2 Department of Neuropsychiatry, Graduate School of Medicine, Nippon Medical School, Tokyo, Japan; 3 Department of Medical Technology, Faculty of Health Sciences, Ehime Prefectural University of Health Sciences, Ehime, Japan; 4 Department of Pharmacology, Graduate School of Medicine, Nippon Medical School, Tokyo, Japan

**Keywords:** Dental anxiety, Fear, Neuroimaging, Auditory stimuli, Brain activity

## Abstract

Dental drilling sounds can induce anxiety in some patients. This study aimed to use functional magnetic resonance imaging (fMRI) to assess the relationship between dental fear and auditory stimuli. Thirty-four right-handed individuals (21 women and 13 men; average age, 31.2 years) were selected. The level of dental fear was assessed using the dental fear survey (DFS). Based on a threshold DFS score > 52, participants were categorized into two groups: dental fear (DF) group (*n* = 12) and control group (*n* = 22). Two types of stimuli were presented in a single session: dental and neutral sounds. Cerebral activation during the presentation of these sounds was evaluated using contrast-enhanced blood oxygenation level-dependent fMRI. In the DF group, dental sounds induced significantly stronger activation in the left inferior frontal gyrus and left caudate nucleus (one-sample *t* test, *P* < 0.001). In contrast, in the control group, significantly stronger activation was observed in the bilateral Heschl’s gyri and left middle frontal gyrus (one-sample *t* test, *P* < 0.001). Additionally, a two-sample *t* test revealed that dental sounds induced a significantly stronger activation in the left caudate nucleus in the DF group than in the control group (*P* < 0.005). These findings suggest that the cerebral activation pattern in individuals with DF differs from that in controls. Increased activation of subcortical regions may be associated with sound memory during dental treatment.

## Introduction

The causes of dental fear (DF) are multifactorial [1]. However, patients with DF can readily identify the specific aspects of dental treatment that trigger fear, including injections, sounds of drills or handpieces, or any number of dental procedures or parts of the dental setting that cause pain [2]. A previous study reported that a past negative experience with tooth drilling was a high-risk factor for DF [3]. Furthermore, factors associated with a past traumatic dental experience that could explain the fear of dental treatment include sound [4]. A recent study reported that individuals’ perceptions of dental sounds (DS) are consistent with their level of DF and sex [5]. Thus, DS can evoke fear and anxiety in patients.

Recently, neuroimaging techniques such as functional magnetic resonance imaging (fMRI) have been introduced to measure cerebral activation induced by visual, cognitive, or perceptual stimuli. Several fMRI studies have reported that audio-visual dental stimuli are associated with increased activity in the brain areas associated with pain [6–9]. Furthermore, a recent systematic review found that audio and/or visual cues mimicking dental treatment consistently activated the brain regions associated with fear in healthy individuals. Moreover, this effect was comparable to that observed in general anxiety [10].

Assessment of brain activity in response to simulated DS stimuli could provide valuable information regarding the effects of dental treatment on stress in patients. However, few fMRI studies have directly investigated cerebral activation in patients with DF during exposure to DS [11]. Actual dental-care situations involve various sounds. Patients never hear only one sound during the treatment. Therefore, in this study, a series of DS was reproduced in a clinical setting. To better understand the relationship between DF and auditory stimuli, we compared cerebral activation associated with DS between individuals with DF and controls using fMRI. We hypothesized that specific regions of the brain would be more reactive to DS in individuals with DF than in controls.

## Materials and methods

### Participants and psychological assessment

The study protocol was approved by the Ethics Committee of Nippon Dental University School of Life Dentistry (NDU-T2013-30) and conformed to the guidelines of the Declaration of Helsinki. Informed consent was obtained from all participants before their inclusion in the study. All participants were given an honorarium (approximately 5000 JPY) after completion of the study. Because few studies have reported the cerebral activation pattern due to DS, the sample size was determined in a pilot study. Using the G*power program (ver.3.1.9.2) [12], with an effect size of 1.2, and *t* tests, we determined that this study required at least 12 participants per group (*α* = 0.05, *β* = 0.20).

Thirty-four right-handed Japanese individuals (21 women and 13 men; age, 19–49 years; average age, 31.2 years [standard deviation (SD) = 9.1]) were recruited from the surrounding community. Their mean educational achievement level was 14.1 years (SD = 2.1). All candidates had undergone dental treatment, had normal vision and hearing, and met all magnetic resonance imaging (MRI) inclusion criteria (no cardiac pacemaker, metallic implants, history of vascular surgery, claustrophobia, or tattoo). Candidates were carefully screened by a clinical expert (MK) using a standardized neuropsychiatric interview process [13]. No participant had a history of psychiatric disorders, significant physical illnesses, head injury, neurological disorders, or substance abuse. Routine MRI was performed to rule out anatomic cerebral abnormalities, and no participant took any medication prior to fMRI. All participants were right-handed according to the Edinburgh Handedness Inventory (EHI) [14]. We defined a right-handed participant as one with an EHI score > 50 according to a previous study [15].

The DF level was assessed using the self-reported Dental Fear Survey (DFS) [16]. A Japanese version of the questionnaire was used to verify the validity and reliability of the test [17]. This questionnaire assesses anxiety-provoking situations associated with dental treatment. It consists of 20 questions scored from 1 to 5, summed to give a total score between 20 and 100. The mean score for the Japanese population has been estimated at 37.4 (SD = 14.1) [17]. Based on an overall score > 52 (Japanese mean score + 1 SD), the participants were categorized into the DF and control groups. Twelve participants (nine women and three men; mean age, 32.3 years; SD = 11.2) with scores ranging from 52 to 82 points were included in the DF group. The remaining 22 participants (12 women and 10 men; mean age, 30.6 years; SD = 7.9) with scores < 52 were included in the control group. Depression and dental anxiety levels were evaluated using the Self-Rating Depression Scale (SDS) [18] and Dental Anxiety Scale (DAS), respectively [19].

### Experimental design and procedure

**Fig. 1:**
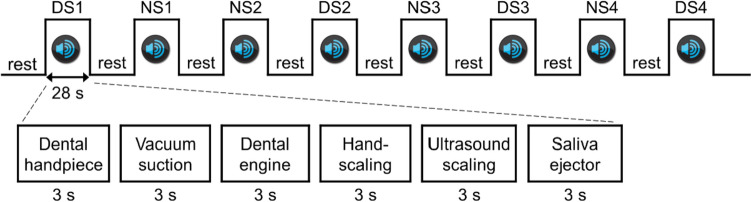
fMRI protocol. A single fMRI session consisted of listening to dental sounds (DS) and neutral sounds (NS). The names indicate the categories of sounds presented. The sequence of the DS–NS or NS–DS blocks was repeated four times, and the duration of each session was 6.4 min

A passive-listening fMRI experiment was conducted using a previously published protocol [20, 21]. In a single session, two types of stimuli were presented: DS and neutral sounds (NS). DS included the sounds of dental drilling using a high-speed dental handpiece, vacuum suction, dental drilling using a low-speed dental engine, hand scaling, ultrasound scaling, and a saliva ejector. The sounds of the French horn or pure tone (2000 Hz), which are not associated with dental treatment, were used as NS. In each listening session, six sounds of that category were presented for 3 s, and the interval between sounds was 2 s, making the duration of each block 28 s. Before each sound category, no sound was presented from the headphones for 20 s (rest condition). Each set lasted 96 s and consisted of two sound conditions (28 s × 2) and two rest conditions (20 s × 2). In the NS condition, the French horn and pure tone were presented alternately. The two stimuli (DS and NS) were presented pseudo-randomly in each set of experiments. Each session consisted of four sets, with a total scanning time of 6.4 min (96 s × 4). (Fig. 1).

All auditory stimuli were presented using Media Studio Pro (version 6.0, Ulead Systems, Inc., Taiwan), and participants listened to the stimuli through headphones connected to an air conductance sound-delivery system (Commancer X6, MRI Audio System, Resonance Technology Inc., Los Angeles, California, USA) [22]. The average sound pressure at the stimulus amplitude was maintained at 80 dB, which is equivalent to the sound pressure of dental drilling using a high-speed dental handpiece during dental treatment.

After completing the session, the participants rated their feelings during exposure to the sounds (six DS and two NS) on a visual analog scale (VAS; 0–100 mm). The following emotional dimensions were assessed: valence, fear, and pain (valence: 0 [very negative], 50 [neutral], and 100 [very positive]; fear and pain: 0 [not at all] and 100 [very strong]) [23].

### Image data acquisition and analysis

Imaging was performed using a Philips 3.0 Tesla MRI system. Functional images of 395 volumes were acquired using T2*-weighted gradient echo-planar imaging (EPI) sequences that are sensitive to blood oxygenation level-dependent (BOLD) contrast. Each volume consisted of 35 transaxial contiguous slices with a slice thickness of 4 mm, covering almost the entire brain (flip angle = 72.5°; echo time = 23 ms; repetition time = 1.6 s; matrix = 52 × 30 × 64; field of view = 208 × 120 × 256) [22].

Data were analyzed using the statistical parametric-mapping software SPM8 (Wellcome Department of Cognitive Neurology, London, United Kingdom), which was run in MATLAB (MathWorks, Natick, Massachusetts, USA). All functional EPI images from each session were realigned to the first volume to correct for the participant’s motion. Images were spatially normalized to the standard space defined by the Montreal Neurological Institute template. In this study, slice thickness was set at 4 mm. For the normalization analysis conducted in SPM8, we utilized a voxel size of 2 × 2 × 2. Following normalization, all scans had a final spatial resolution of 2 × 2 × 2 mm^3^. Functional images were smoothed using a 3-D isotropic Gaussian kernel with a full width at half maximum of 8 mm. A high-pass filter was applied to the fMRI time series to remove low-frequency noise and enhance the temporal signal-to-noise ratio. Hemodynamic changes during each condition were analyzed using a general linear model combined with boxcar functions convoluted with hemodynamic response functions. Voxel-by-voxel statistical parametric maps were constructed for each *t* statistic. The *t* values were transformed into a unit normal distribution to obtain *z* scores.

Models of the two stimuli (DS and NS) were created using a blocked design for fMRI experiments. First, to investigate the effect of DF on cerebral activation during auditory processing, cerebral activation during the two stimuli was analyzed. Next, to clarify cerebral activation during exposure to DS, cerebral activation in the DS minus NS contrast was examined based on previous studies [20, 21].

Group analysis (2nd-level analysis in SPM8) was performed on the data of 22 participants in the control group and 12 participants in the DF group using a random-effects model on a voxel-by-voxel basis. Two trials (DS and NS) were presented for each explanatory variable. Each explanatory variable was convoluted with a standard hemodynamic response function taken from SPM8 to account for the hemodynamic response lag. First, we analyzed the one-sample *t* test for cerebral activation of the control and DF groups, respectively. Second, we evaluated the difference in the mean cerebral activation between the DF and control groups using a two-sample *t* test, and *t* statistics were calculated to compare the two trials. Additionally, we analyzed the one-sample *t* test for cerebral activation in all 34 participants. Five regions of interests (ROIs) were identified from the peaked activations in this analysis. Cerebral activation at the ROIs was investigated in each group. For the main effect of task, ROIs were defined as sphere voxels of 10 mm radius from the coordinates of the peak voxel of activation.

Demographic data for the DF and control groups were compared using Student’s *t* test and Fisher’s exact test. A two-way analysis of variance was used to analyze the subjective ratings between the two groups for DS and NS. Statistical significance was set at *P* < 0.05, and all analyses were conducted using IBM SPSS Statistics 21 software (IBM Japan, Tokyo, Japan).

## Results

### Participants characteristics and behavioral data

The baseline characteristics of the study participants are shown in Table 1. The sex ratio, age, duration of education, EHI score, and SDS score were not significantly different between the groups. Significant differences were observed only in the DAS and DFS scores between the groups, which were related to the DF level (*P* = 0.002 and *P* < 0.001, respectively).

**Table 1:** Characteristics of the participants

	DF group (*n* = 12)	Control group (*n* = 22)	*P* value*
Sex ratio (F/M)	9/3	12/10	0.29
Age (years)	32.3 ± 11.2	30.6 ± 7.9	0.63
Education (years)	13.8 ± 1.6	15.2 ± 2.3	0.08
EHI score	92.7 ± 17.1	94.1 ± 9.8	0.75
SDS score	37.5 ± 8.0	38.5 ± 8.6	0.74
DAS score	11.6 ± 2.0	8.2 ± 3.2	0.002
DFS score	62.9 ± 10.3	31.3 ± 8.9	<0.001

Data are shown as Mean ± standard deviation

*DF* dental fear; *EHI* Edinburgh handedness inventory; *SDS* self-rating depression scale; *DAS* dental anxiety scale; *DFS* dental fear survey

^*^t test and Fisher’s exact test

The mean subjective ratings for DS in each group are shown in Table 2. The main effect of group was significant for ratings of valence, fear, and pain (valence, *F* (1, 192) = 22.803, *P* < 0.001; fear, *F* (1, 192) = 18.519, *P* < 0.001; pain, *F* (1, 192) = 7.808, *P* = 0.006). The main effect of sound was significant for ratings of valence, fear, and pain (valence, *F* (5, 192) = 2.353, *P* = 0.042; fear, *F* (5, 192) = 3.946, *P* = 0.002; pain, *F* (5, 192) = 6.101, *P* < 0.001). The interaction effect (group × sound) was not significant for all ratings.

**Table 2:** Subjective ratings for dental sounds

	DF group (*n* = 12)	Control group (*n* = 22)	Significant effects*
Valence			
D1	9.8 ± 10.7	21.8 ± 22.2	
D2	22.3 ± 14.2	38.1 ± 20.1	
D3	19.4 ± 16.5	31.1 ± 19.2	G, S
D4	22.6 ± 16.3	26.1 ± 18.6	
D5	12.7 ± 11.8	29.2 ± 19.7	
D6	18.7 ± 13.7	35.2 ± 20.0	
Fear			
D1	83.5 ± 21.3	54.8 ± 36.0	
D2	48.2 ± 27.7	45.5 ± 32.5	
D3	59.1 ± 32.5	41.5 ± 31.0	G, S
D4	61.1 ± 26.6	35.0 ± 32.1	
D5	67.7 ± 25.8	49.1 ± 34.9	
D6	48.5 ± 31.0	25.6 ± 26.3	
Pain			
D1	75.0 ± 28.8	52.9 ± 40.8	
D2	27.8 ± 25.3	27.6 ± 28.8	
D3	46.0 ± 32.7	31.6 ± 31.1	G, S
D4	55.5 ± 29.5	40.3 ± 31.9	
D5	53.6 ± 37.0	42.5 ± 37.4	
D6	34.3 ± 31.0	18.7 ± 22.5	

Data are shown as Mean ± standard deviation

*DF* dental fear; *D1* dental drilling with high-speed dental handpiece; *D2* vacuum suction; *D3* dental drilling with low-speed dental engine; *D4* hand-scaling; *D5* ultrasound scaling; *D6* saliva ejector; *N1* French horn; *N2* pure tone

^*^Two-way analysis of variance. Significant effects denote main effect for G (group) and S (sound)

Table 3 shows the mean subjective ratings for NS in each group. The significant main effect of group was not observed (valence, *P* = 0.43; fear, *P* = 0.24; pain, *P* = 0.58). The main effect of sound was only significant for ratings of valence (valence, *F* (1, 64) = 4.345, *P* = 0.041; fear, *P* = 0.24; pain, *P* = 0.67). The interaction effect (group × sound) was not significant for all ratings.

**Table 3:** Subjective ratings for neutral sounds

	DF group (*n* = 12)	Control group (*n* = 22)	Significant effects*
Valence			
N1	44.3 ± 21.1	51.2 ± 18.9	S
N2	36.5 ± 16.4	37.6 ± 22.6	
Fear			
N1	28.6 ± 29.0	12.8 ± 21.6	n.s.
N2	29.3 ± 23.0	28.7 ± 33.3	
Pain			
N1	16.6 ± 27.2	10.6 ± 20.8	n.s.
N2	16.3 ± 23.2	15.8 ± 21.6	

Data are shown as Mean ± standard deviation

*DF* dental fear; *D1* dental drilling with high-speed dental handpiece; *D2* vacuum suction; *D3* dental drilling with low-speed dental engine; *D4* hand-scaling; *D5* ultrasound scaling; *D6* saliva ejector; *N1* French horn; *N2* pure tone

^*^Two-way analysis of variance. Significant effects denote main effect for S (sound). n.s.: not significant

### fMRI data

**Fig. 2:**
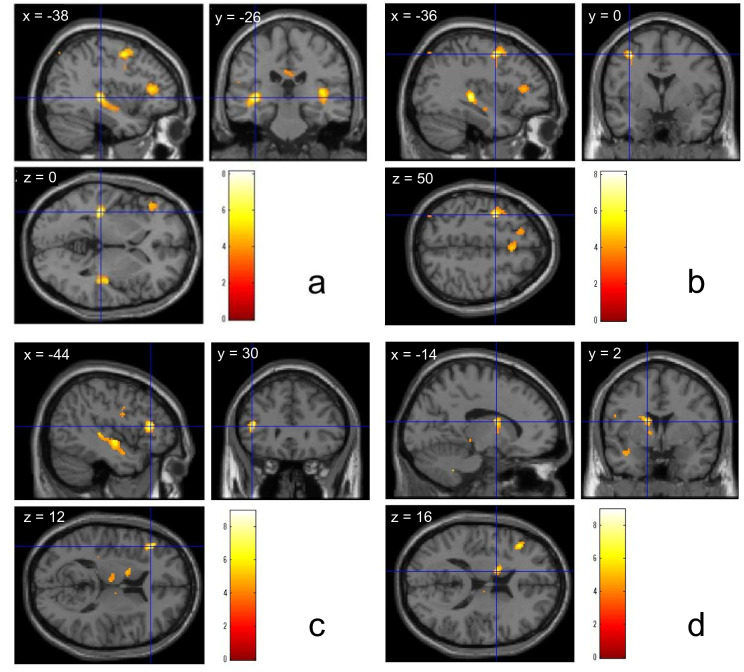
Activated regions in the DS–NS contrast. In the control group, DS induce significantly stronger activation in the left and right Heschl’s gyri (**a**) and left middle frontal gyrus (**b**) compared to NS (one-sample *t* test). In the DF group, significant brain activity is observed in the left inferior frontal gyrus (**c**) and left caudate nucleus (**d**) (one-sample *t* test, statistical height threshold was set at an uncorrected *P* value of <0.001, extent threshold was set at *k* > 10 voxels, and cluster-level FWE correction was applied with a *P* value of <0.05). DS, dental sounds; NS, neutral sounds; DF, dental fear; FWE, family wise error

**Fig. 3:**
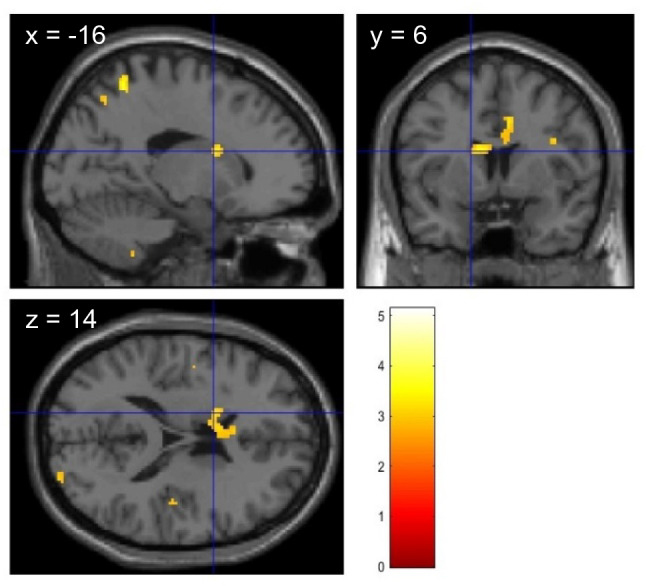
Activated regions in the DS–NS contrast (DF group vs. control group). DS induce significantly greater activation of the left caudate nucleus in the DF group than in the control group (two-sample *t* test, statistical height threshold was set at an uncorrected *P* value of <0.005, extent threshold was set at *k* > 10 voxels, and cluster-level FWE correction was applied with a *P* value of <0.05). DS, dental sounds; NS, neutral sounds; DF, dental fear; FWE, family wise error

The control group showed significantly stronger activation in the bilateral Heschl’s gyri (HG) (Fig. 2a), left inferior frontal gyrus (IFG), and left middle frontal gyrus (MFG) (Fig. 2b) during exposure to DS than during exposure to NS (one-sample* t* test, statistical height threshold: *P* < 0.001 uncorrected, extent threshold: *k* > 10 voxels, cluster-level FWE correction: *P* < 0.05). The DF group showed significantly stronger brain activity in the left HG, left IFG (Fig. 2c), and left caudate nucleus (Fig. 2d) during exposure to DS than during exposure to NS (one-sample *t* test, statistical height threshold: *P* < 0.001 uncorrected, extent threshold: *k* > 10 voxels, cluster-level FWE correction: *P* < 0.05). Additionally, significantly stronger activation was observed in the left caudate nucleus during exposure to DS in the DF group than in the control group (Fig. 3) (two-sample *t* test, statistical height threshold: *P* < 0.005 uncorrected, extent threshold: *k* > 10 voxels, cluster-level FWE correction: *P* < 0.05). Table 4 shows the brain regions activated in the DS–NS contrast in each group and the differences between the groups. The coordinates of significant BOLD responses in the ROI analysis for each group are presented in Table 5.

**Table 4:** Activated brain regions in response to DS–NS

Group	Brain regions	MNI coordinates			*z* value	Voxel *P*
		*x*	*y*	*z*		
C	Heschl’s gyrus L	−38	−26	0	4.98	0.001*
	Heschl’s gyrus R	38	−28	8	4.43	0.001*
	Inferior frontal gyrus L	−52	16	22	4.69	0.001*
	Middle frontal gyrus L	−36	0	50	5.42	0.001*
DF	Heschl’s gyrus L	−42	−10	−6	4.09	0.001*
	Inferior frontal gyrus L	−44	30	12	4.73	0.001*
	Caudate nucleus L	−14	2	16	4.33	0.001*
DF > C	Caudate nucleus L	−16	6	14	3.14	0.005**

*DS* dental sounds; *NS* neutral sounds; *MNI* Montreal Neurological Institute; *C* control group; *DF* dental fear group; *L* left; *R* right; *FWE* family wise error

^*^Statistical height threshold was set at an uncorrected *P* value of <0.001, extent threshold was set at *k* > 10 voxels, and cluster-level FWE correction was applied with a *P* value of <0.05

^**^Statistical height threshold was set at an uncorrected *P* value of <0.005, extent threshold was set at *k* > 10 voxels, and cluster-level FWE correction was applied with a *P* value of <0.05

**Table 5:** Coordinates for significant BOLD responses for the ROI analysis

Brain regions	DS > NS					
	Control group			DF group		
	*x*	*y*	*z*	*x*	*y*	*z*
Heschl’s gyrus L	−38	−26	0	−42	−10	−6
Heschl’s gyrus R	38	−28	8		–	
Inferior frontal gyrus L	−52	16	22	−44	30	12
Middle frontal gyrus L	−36	0	50		–	
Caudate nucleus L		–		−14	2	16

*BOLD* blood oxygenation level dependent; *ROI* regions of interest; *DS* dental sounds; *NS* neutral sounds; *DF* dental fear group; *L* left, *R* right

## Discussion

In this study, we examined the subjective ratings and brain activity in individuals with and without DF in response to simulated DS stimuli. The data from subjective ratings indicated that individuals with DF exhibited a higher fear response to DS than those without DF. In addition, brain activity was stronger in the left caudate nucleus after exposure to DS. Therefore, our hypothesis that specific brain regions would be more reactive to DS in individuals with DF than in controls was accepted.

An epidemiological study reported that among various dental procedures, invasive dental stimuli, such as dental drilling and surgical procedures, were rated as the most anxiety-evoking stimuli, and the rank order of these stimuli appeared to be independent of sex, age, ethnicity, and the dental anxiety level [24]. In the present study, compared to the control group, the DF group showed significantly more negative valence ratings for DS. Although the results were not examined separately according to sex, the DF group showed a significantly stronger fear of DS than the control group. There was no significant difference in the valence for NS between the two groups. A previous study reported that subjective DS ratings vary according to the level of DF and sex, with women who have DF potentially exhibiting a heightened negative sensitivity to dental drilling and vacuum-suction sounds [5]. However, due to the small sample size in the present study, we were unable to replicate the findings of the previous study.

The HG, also called the transverse temporal gyrus, is part of the human auditory cortex [25]. It is located in the temporal plane inside the lateral sulcus and is sometimes treated as a part of the superior temporal gyrus. HG plays an important role in understanding speech and encoding auditory features during perception [26]. A previous study indicated that the bilateral superior temporal gyri are involved in the perception and production of speech sounds [27]. In our study, the control group showed significantly stronger bilateral HG activation during exposure to DS. This suggests that DS may have been perceived as a normal auditory stimulus by participants without DF. However, the DF group showed significantly stronger activation of the left HG. The functional processing of acoustic attributes is not different between the left and right HGs [28]. Our results suggest that the perception of DS may differ patients with and without DF.

Studies on speech perception and working memory have shown that the left IFG is an important area related to the multisensory integration of audiovisual perception [29]. Furthermore, the left IFG and its adjacent areas are integral parts of both speech perception and speech production networks [30]. A previous study emphasized the role of sensorimotor brain regions (the left IFG and insula) in speech perception [31]. Recent studies in typically developing adults suggest that the role of the left IFG and its adjacent areas in speech perception is particularly important for speech recognition in noisy conditions [32, 33]. In our study, significant brain activity was observed in the left IFG in both groups. In other words, these results suggest that the participants in both groups were trying to analyze the type of sound. Thus, left IFG activation could be a neural marker of effortful listening [33].

Activation of the left MFG has consistently been linked to working memory, social information processing and perception, emotional stimulus processing, and emotion regulation [34]. These cognitive functions of the left MFG are consistent with the cognitive factors associated with rumination and trait rumination [34]. Previous studies have demonstrated that anger rumination is correlated with activation of the prefrontal cortex, which is responsible for evaluating affective stimuli [35, 36]. In this study, individuals in the control group showed stronger activation in the left MFG during exposure to DS. Since no subjective ratings of anger or autonomic responses to DS were obtained, the degree of anger elicited by DS remains unclear. However, participants in the control group may have processed emotional stimuli and regulated the emotions elicited by DS.

Notably, in the DF group, significantly stronger activation was observed in the left caudate nucleus during exposure to DS. Recent studies have indicated that the basal ganglia, including the caudate nucleus, are involved in learning and memory [37]. A previous study evaluating the response to visual dental stimuli found significant activations in the dorsolateral prefrontal cortex and caudate nucleus in men and women with dental phobia, respectively [38]. A meta-analysis of neuroimaging studies reported that women showed higher activation of negative emotions in the left caudate nucleus than men [39]. The basal ganglia can be considered parts of the pain regulatory system, including emotional, autonomic, and cognitive responses to nociceptive stimuli [38]. Furthermore, women more strongly avoid or give up treatment and exhibit enhanced pain memory for dental treatment than men [40, 41]. Owing to the small sample size of this study, we were unable to examine sex differences in the neural mechanisms of participants with DF. We should acknowledge that sex may have acted as a confounding factor, influencing the observed cerebral activation in response to DS. To address this, future studies should aim to increase the sample size and conduct separate analyses for each sex.

Hilbert et al. [11] used DFS, similar to the present study, to recruit participants and found that the dental phobia group showed a greater response in the insular cortex than healthy controls when exposed to the sound of a dental drill. Similarly, Yeung et al. [6] demonstrated a correlation between DF levels and insular cortical activation. A previous study has identified the anterior and posterior insular cortices as areas of brain activation that anticipate pain [42]. These findings suggest that individuals with severe DF may recall pain on exposure to DS.

Although subjective ratings of pain in response to DS differed significantly between the two groups, no activation of the insular cortex was observed in the DF group in this study. This finding is inconsistent with the findings in previous studies that demonstrated activation of the pain domain after exposure to DS. This may be due to the lower DF level among participants in the DF group in our study compared to that in previous studies. Hilbert et al. [11] compared patients with DFS scores ≥ 72 with those with DFS scores ≤ 33. In contrast, the cut-off score in the present study was 52. Several studies have concluded that patients with a high DF report more pain than those with a low DF [43]. Another possibility is that participants did not experience severe pain during their previous dental treatments. However, participants’ past painful experiences were not identified in this study. Another reason is that this study reproduced six DS, including the sound of the dental drill, vacuum suction, and other instruments. This may have diluted the effect of the dental drill sound, which caused the highest fear levels.

The findings in this study showed that individuals with high DF react differently to DS than those without DF. Therefore, sound control during dental procedures is crucial for successful, smooth, and safe treatment. Masking DS with headphones or earphones or listening to one’s favorite music during dental treatment can effectively alleviate anxiety and pain [44]. In addition, patient care may require the use of less invasive and soundless instruments instead of dental drills, such as the spoon excavator, which is frequently used in pediatric dentistry.

This study had some limitations. First, the sample size was small. Therefore, we were unable to examine sex differences in the neural mechanisms underlying DF. In the future, the sample size should be increased and DF should be examined separately for men and women. Second, this study included a series of DS, based on the actual dental-treatment conditions. Therefore, it was not possible to examine the differences in cerebral activation caused by the sound of each instrument, and further studies are needed to clarify these differences. Third, although levels of DF and dental anxiety were assessed, past dental trauma and pain catastrophizing were not investigated. The effects of these variables on brain activity should be evaluated in future studies. Additionally, future studies should be designed considering the possibility that the block design itself may lead to immediate habituation and prevent the discovery of activity patterns.

Within the limitations of this study, our findings indicate that brain activity patterns observed in individuals with DF differ from those without DF. Increased activity in the subcortical region may be related to memorization of sounds associated with dental treatment. However, further studies are needed to confirm this hypothesis.

## Data Availability

The datasets utilized in the current study are available from the corresponding author upon reasonable request.
